# Pathogenic variants in SMARCA1 cause an X-linked neurodevelopmental disorder modulated by NURF complex composition

**DOI:** 10.21203/rs.3.rs-3317938/v1

**Published:** 2023-09-29

**Authors:** David Picketts, Ghayda Mirzaa, Keqin Yan, Raissa Relator, Sara Timpano, Binnaz Yalcin, Stephan Collins, Alban Ziegler, Emily Pao, Nora Oyama, Elise Brischoux-Boucher, Juliette PIARD, Kristin Monaghan, Maria Guillen Sacoto, William Dobyns, Kristen Park, Daniel Fernández-Mayoralas, Alberto Fernández-Jaén, Parul Jayakar, Alfredo Brusco, Vincenzo Antona, Elisa Giorgio, Malin Kvarnung, Bertrand Isidor, Solène Conrad, Benjamin Cogné, Wallid Deb, K.E. Stuurman, Katalin Sterbova, Noor Smal, Sarah Weckhuysen, Renske Oegema, Micheil Innes, Maeson Latsko, Tawfeg Ben-Omran, Rebecca Yeh, Michael Kruer, Somayeh Bakhtiari, Antigone Papavasiliou, Sébastien Moutton, Sophie Nambot, Sirisak Chanprasert, Sarah Paolucci, Kait Miller, Barbara Burton, Katherine Kim, Emily O’Heir, Zandre Bruwer, Kirsten Donald, Tjitske Kleefstra, Amy Goldstein, Brad Angle, Kelly Bontempo, Peter Miny, Pascal Joset, Florence Demurger, Emma Hobson, Lewis Pang, Lori Carpenter, Dong Li, Dominique Bonneau, Bekim Sadikovic

**Affiliations:** Ottawa Hospital Research Institute; Seattle Children’s Hospital; Ottawa Hospital Research Institute; London Health Sciences Centre; Ottawa Hospital Research Institute; Inserm; INSERM UMR-S 1231, University of Bourgogne Franche-Comté; University Hospital of Angers; Seattle Children’s Research Institute; Seattle Children’s Research Institute; Université de Franche-Comté; CHU Besançon; GeneDx; GeneDx, Gaithersburg, MD; University of Minnesota; University of Colorado Denver School of Medicine; Hospital Universitario Quirónsalud; Department of Pediatrics and Neurology, Hospital Universitario Quirónsalud, School of Medicine, Universidad Europea de Madrid; Division of Genetics and Metabolism, Nicklaus Children’s Hospital; University of Turin; University of Palermo; University of Pavia; Karolinska Institutet; CHU de Nantes; Nantes Université; CHU Nantes; Nantes Université; Department of Clinical Genetics, Erasmus University Medical Center; Charles University and Motol Hospital; VIB Center for Molecular Neurology; VIB Center for Molecular Neurology; University Medical Center Utrecht; University of Calgary; The Steve and Cindy Rasmussen Institute for Genomic Medicine; Hamad Medical Corporation; Boston Children’s Hospital; Phoenix Children’s Hospital; University of Arizona College of Medicine; IASO Children’s Hospital; CHU François Mitterrand; Centre de Génétique et Centre de référence «Anomalies du Développement et Syndromes Malformatifs», Hôpital d’Enfants, Centre Hospitalier; University of Washington; University of Washington; University of Washington School of Medicine; Northwestern University Feinberg School of Medicine; Northwestern University Feinberg School of Medicine; Broad Institute of MIT and Harvard; University of Cape Town; Division of Developmental Paediatrics, Department of Paediatrics and Child Health, Red Cross War Memorial Children’s Hospital, Klipfontein Road/Private Bag, Rondebosch, 7700/7701, Cape Town, South A; Radboud University Medical Centre; Children’s Hospital of Pittsburgh of UPMC; Advocate Children’s Hospital; Advocate Children’s Hospital; University Hospital Basel; University Hospital Basel; Institute of Genetics & Development of Rennes; Leeds Teaching Hospitals Trust; Royal Devon and Exeter; St Francis Health Systems; The Children’s Hospital of Philadelphia; Department of Biochemistry and Genetics, University Hospital of Angers, F-49000

**Keywords:** SMARCA1, brain overgrowth, exome sequencing, epigenetics, NURF complex

## Abstract

Pathogenic variants in ATP-dependent chromatin remodeling proteins are a recurrent cause of neurodevelopmental disorders (NDDs). The NURF complex consists of BPTF and either the SNF2H (*SMARCA5*) or SNF2L (*SMARCA1*) ISWI-chromatin remodeling enzyme. Pathogenic variants in *BPTF* and *SMARCA5* were previously implicated in NDDs. Here, we describe 40 individuals from 30 families with *de novo* or maternally inherited pathogenic variants in *SMARCA1*. This novel NDD was associated with mild to severe ID/DD, delayed or regressive speech development, and some recurrent facial dysmorphisms. Individuals carrying *SMARCA1* loss-of-function variants exhibited a mild genome-wide DNA methylation profile and a high penetrance of macrocephaly. Genetic dissection of the NURF complex using *Smarca1, Smarca5*, and *Bptfsingle* and double mouse knockouts revealed the importance of NURF composition and dosage for proper forebrain development. Finally, we propose that genetic alterations affecting different NURF components result in a NDD with a broad clinical spectrum.

## Introduction

The ATP-dependent chromatin remodeling family consists of proteins that contain a well conserved SNF2-domain that can be further subdivided into the SWI/SNF, ISWI, CHD, and INO80 sub-families, distinguished by additional chromatin interaction motifs and distinct protein interacting partners (1). These remodeling complexes utilize the energy from ATP hydrolysis to reposition nucleosomes and are critical for DNA replication, transcription, and DNA repair (2). Many have been implicated in human diseases, including various cancers and a broad range of neurodevelopmental disorders (NDDs) (3–5). The best characterized is the SWI/SNF family, comprising BRG1 and BRM that are interchangeable subunits within a large 12–15 protein complex known primarily as the BRG1-associated factor (BAF) complex. Pathogenic variants in the genes encoding BRG1 (*SMARCA4*) and BRM (*SMARCA2*) are the cause of Coffin-Siris 4 (CS4, MIM 614609) and Nicolaides-Baraitser (MIM 601358) syndromes, respectively (6, 7). Additionally, mutations in the genes encoding most other BAF subunits also cause similar features, leading to the suggestion that a defective BAF complex drives a broad neurodevelopmental spectrum with autistic features at the mild end, classic Coffin-Siris syndrome in the middle, and Nicolaides-Baraitser syndrome representing the most severe disease (3). The proteins encoded by the CHD and INO80 gene families also interact within large multiprotein complexes and several family members from each group (CHD2, CHD3, CHD4, CHD7, CHD8; INO80, SRCAP) have been implicated in distinct NDDs (8).

The mammalian ISWI family consists of two genes, *SMARCA1* and *SMARCA5* which encode the SNF2L and SNF2H remodeling proteins respectively, that are orthologs of the *Drosophila* ISWI protein. Both mammalian ISWI proteins contain the conserved SNF2-domain common to all remodeler families and a HAND-SANT-SLIDE (HSS) domain that interacts with histone H4 tails and linker DNA (9). While most remodeling complexes contain 10–15 subunits, the ISWI complexes are heterodimeric, pairing one of the ATPase subunits with either a member of the ~180-kDa bromodomain adjacent to zinc finger (BAZ) transcription factor family, or a larger protein (>300 kDa) with related chromatin domains (BPTF, CECR2, or RSF1). Initial purification of the ISWI complexes suggested that each ATPase protein bound a subset of regulatory proteins; SNF2L interacted within the NURF (BPTF) and CERF (CECR2) complexes while the ACF/CHRAC (BAZ1A), WICH (BAZ1B), NoRC (BAZ2A), and RSF (RSF1) complexes contained SNF2H (10). Note that the CHRAC complex differs from the ACF complex by the co-purification of two additional auxiliary proteins (CHRAC15/17); the NURF complex is also multimeric containing the Rb-associated proteins RbAp46 and RbAp48 (11). More recently, BAZ2B was shown to interact with both ISWI proteins, with further work showing that SNF2H and SNF2L proteins are interchangeable with all regulatory subunits thereby suggesting the existence of 16 distinct ISWI complexes (12).

Biochemical studies indicate that ISWI protein complexes are involved in nucleosome assembly and spacing, replication of heterochromatin, organization of higher order chromosome structure, and transcriptional regulation (13). The ACF1 complex is involved in general nucleosome assembly, while RSF1 assembles centromeric chromatin and NoRC mediates heterochromatin formation to silence rDNA repeats (14–16). ISWI proteins also regulate transcription through the maintenance of nucleosome free regions (NFRs). In *Drosophila*, ISWI genome-wide binding data demonstrated that the protein is located in genic and intergenic regions with peak binding 300 bp downstream of the TSS where it helps position the first two nucleosomes (17). Multiple studies have shown roles for the NURF complex in target gene expression during cell differentiation through interactions with a wide range of transcription factors and the H3K4me3 histone mark (11, 18–24). The ACF and WICH complexes are important for replication through heterochromatin during S-phase and for repair of DNA breaks (25–31). Specific functions have not been attributed to CERF or the BAZ2B complexes. Taken together, these disparate functions suggest that there are multiple mechanisms by which ISWI complexes could alter neurodevelopment if specific components were mutated and not functioning optimally.

In general, the human ISWI genes are ubiquitously expressed with *SMARCA1* expressed in most tissues while *SMARCA5* was detected in all tissues examined (32). In the developing mouse brain, ISWI transcript and protein levels show a consistent pattern with *Smarca5* enriched earlier when progenitor cells are abundant, and *Smarca1* elevated later and predominantly in terminally differentiated neurons (33–35). ISWI gene inactivation in mice supports distinct roles for the ISWI proteins. *Smarca5* conditional knockout mice are reduced in size and have severe defects in progenitor proliferation resulting in forebrain hypoplasia or impaired postnatal growth of the cerebellum depending on the Cre-driver line used to ablate the gene (33, 36). Indeed, the *Smarca5* mouse models are representative of the phenotype in individuals with *SMARCA5* germline pathogenic variants who present with developmental delay, short stature, and microcephaly (37). In contrast, progenitor differentiation is delayed in *Smarca1* mutant mice (Ex6DEL) leading to increased neuronal production and mice with larger brains (38). Although a specific syndrome has not been associated with *SMARCA1* germline variants, several whole genome sequencing screens of large patient cohorts including individuals with intellectual disability/developmental delay (ID/DD) have identified *SMARCA1* pathogenic variants in a small number of families (39–41). Other pathogenic variants within genes encoding ISWI family interactors that result in neurodevelopmental disorders include *BAZ1B*, which lies within the commonly deleted region causing Williams-Beuren syndrome (MIM 194050) (42, 43); *BAZ2B* (44); and *BPTF*, as the cause of the NEurodevelopmental Disorder with Dysmorphic Facies and distal Limb anomalies (NEDDFL) syndrome (MIM 617755) (45, 46).

Here, we report the identification of 40 individuals from 30 families with de novo or inherited germline variants within the *SMARCA1*gene associated with variable degrees of developmental delay and speech regression. In addition, we demonstrate the importance of the NURF complex to murine neocortical development and suggest that altered composition and dosage of this complex contributes to clinical variability.

## Results

### Identification of SMARCA1 genetic variants

**Figure 1:**
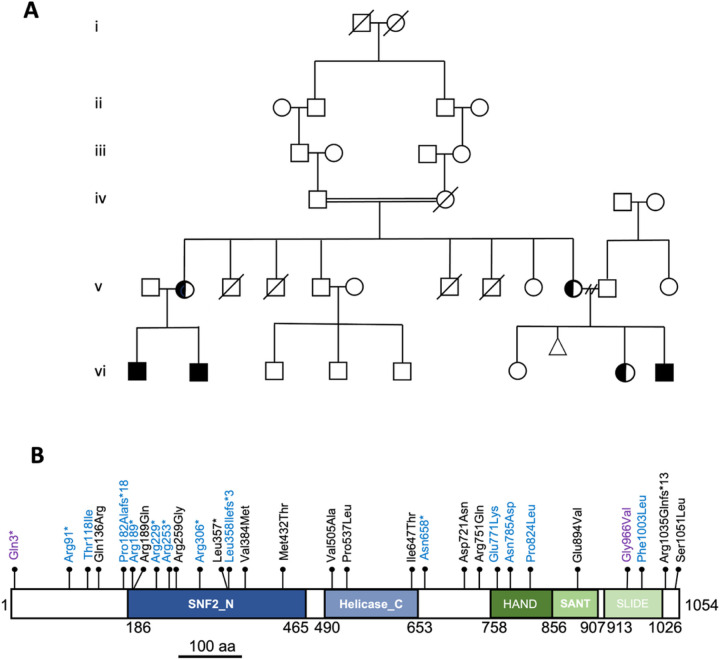
Identification and location of pathogenic variants in the *SMARCA1* gene. A) Six generation pedigree of the index family carrying the c.271C>T [p.Arg91*] variant within *SMARCA1*. Genetic testing also included the maternal uncle (v5) and grandfather (iv1) who were both negative for the variant. B) Schematic diagram highlighting the position of the pathogenic variants identified and the functional domains encoded by *SMARCA1*. Blue text denotes variants with macrocephaly, purple text refers to previously described variants that were not identified in this study. Numbers refer to amino acid position. The motifs comprising the SNF2 ATPase/helicase domain and HSS domain are highlighted in blue and green, respectively.

The initial characterization of a large six-generation family (Figure 1A) containing 4 individuals with mild to severe ID/DD and brain overgrowth identified a hemizygous nonsense variant (c.271C>T [p.Arg91*]) in the *SMARCA1* gene (NM_003069.4) in the proband (vi1), his brother (vi2) and two maternal cousins (vi8 and vi9). Both mothers (v2 and v10) of the affected individuals also tested positive for the variant while the paternal grandfather (iv1) and maternal uncle (v5) were negative. An additional 29 families were ascertained in part through the MatchMaker Exchange network (47) for a total of 40 individuals with apparent non-mosaic hemizygous *SMARCA1* variants. A total of 27 unique variants were identified including a second unrelated family with the same variant as the index family (Figure 1B, Table 1). Loss-of-function (LOF) variants included seven nonsense variants (c.271C>T [p.Arg91*], c.565C>T [p.Arg189*], c.685C>T [p.Arg229*], c.757C>T [p.Arg253*], c.916C>T [p.Arg306*], c.1070T>G [p.Leu357*], c.1971dup [p.Asn658*]), and three frameshifting insertions/deletions (indels) affecting coding exons (c.543dupG [p.Pro182Alafs*18], c.1071dupA [p.Leu358Ilefs*3], c.3103–3106del [p.Arg1035Glnfs*13]). Seventeen missense variants (c.353C>T [p.Thr118Ile], c.407A>G [p.Gln136Arg], c.566G>A [p.Arg189Gln], c.775C>G [p.Arg259Gly], c.1150G>A [p.Val384Met], c.1295T>C [p.Met432Thr], c.1514T>C [p.Val505Ala], c.1610C>T [p.Pro537Leu], c.1940T>C [p.Ile647Thr], c.2161G>A [p.Asp721Asn], c.2161G>A [p.Arg751Gln], c.2311G>A [p.Glu771Lys], c.2353A>G [p.Asn785Asp], c.2471C>T [p.Pro824Leu], c.2681A>T [p.Glu894Val], c.3007T>C [p.Phe1003Leu], c.3152C>T [p.Ser1051Leu]) were also identified. The p.Val384Met and p.Pro537Leu missense variants were each identified in multiple unrelated families. The c.7C>T [p.Gln3*] and c.2897G>T [p.Gly966Val] variants were previously reported in isolated cases with neurodevelopmental disorders, but absent in our cohort (40, 41).

Within the 30 index cases, 22 had a maternally inherited variant, three variants occurred *de novo*, and for five cases the segregation was not determined, although 4 of these were presumably maternal inheritance. All *SMARCA1* variants reported in this study were absent in gnomAD v2.1.1 with the exception of c.1150G>A [p.Val384Met] and c.1295T>C [p.Met432Thr], the former present once (single hemizygote allele) and the latter twice (one heterozygote and one hemizygote allele), both with very low allele frequencies (1.645x10^−5^ and 1.099x10^−5^, respectively). The p.Val384Met variant was also previously identified within a pedigree of familial schizophrenia that co-segregated with a variant in the *AVEN* gene, drawing into question the causality of this variant (39). Nonetheless, all missense variants were predicted as deleterious using multiple bioinformatic algorithms (SIFT, Polphen-2, MutationTaster, and CADD).

The highly conserved ATPase domain (comprising SNF2_N and Helicase_C motifs) and the HSS histone interaction module of *SMARCA1* are critical for nucleosome remodeling activity (48). Eighteen of the 27 unique variants identified resided within these two functional domains (13 in ATPase, 5 in HSS) with an additional previously reported case (p.Gly966Val) also located in the HSS domain. The remaining variants were located either adjacent to these domains or near the C-terminus of the protein coding sequence (Figure 1B). Nonetheless, the identification of 10 individuals with nonsense or frameshift LOF variants are strongly suggestive of disease causation.

### Clinical findings of individuals with SMARCA1 variants

Affected individuals included 31 males and 9 females, aged 2 to 48 years at their last clinical assessment. The affected cohort showed a broad range of clinical features that are summarized in Table 1. Most individuals for whom clinical data were available presented with ID/DD (80%; 31/39) and speech delay or speech regression (85%; 34/40). Increased head circumference (OFC 2 SD) and/or reported macrocephaly/brain overgrowth (45%; 17/38) was identified in the index family and reported in eight individuals with LOF variants (Figure 1B; Table 1). However, three individuals with LOF variants without brain overgrowth and five individuals with missense variants with brain overgrowth phenotype suggest that this feature was more complex and not restricted to LOF variants. Increased height ( 2SD; 29%; 10/34) and weight ( 2SD; 31%; 10/32) at the time of the last exam were reported slightly less frequently arguing against a generalized overgrowth syndrome (Table 1; Suppl. Figure 1). Other commonly reported clinical features included motor delay (25/39; 64%), seizures/epilepsy (24%; 9/38), and hypotonia (42%; 16/38). Many individuals (23/40; 58%) were diagnosed with behavioral difficulties (ASD, ADHD, aggression) and in some instances (9/25) this was linked to regression in speech and mental development. Dysmorphic facial features (58%; 23/40) were also commonly reported although clinical descriptions were variable (Table 1). Photographs from eleven affected individuals show that frontal bossing, a long face, slanted palpebral fissures, a flat nasal root with a short bulbous nose, anteverted nares, and a thin upper lip were relatively common features of the cohort.

### Genomic DNA methylation profiles associated with SMARCA1 variants

Given the broad phenotypic features of individuals with *SMARCA1* variants we explored whether they exhibit a distinguishing methylation profile in their peripheral blood DNA. Genome-wide DNA methylation analysis was performed on DNA derived from peripheral blood from 19 individuals and further stratified by gender. Initial analyses of all samples did not return any favorable outcome. The SMARCA1_M sub-cohort consisted of 12 male individuals with *SMARCA1* variants, two of whom had lymphoblast cell line (LB-line) replicate samples which were also used for investigating profile clustering (SMARCA1_M_rep). An additional male sample was also used for test clustering. On the other hand, the SMARCA1_F sub-cohort consisted of seven female individuals carrying *SMARCA1* variants with or without phenotypic presentation, three of which have LB-line replicate samples (SMARCA1_F_rep) which were only used for the unsupervised clustering.

**Figure 3:**
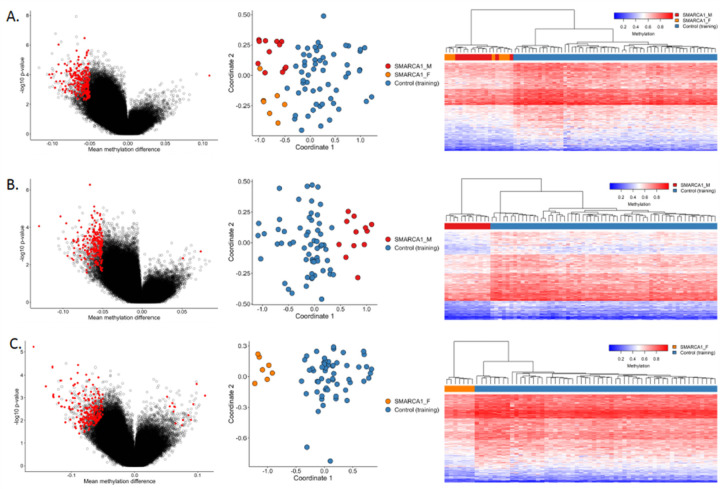
Identified methylation profiles for individuals with *SMARCA1* variants. Volcano plots (left) show mean methylation difference of cases and controls plotted against the −log10(p) values and highlight the selected differential probes for the distinct profiles of (A) *SMARCA1* cohort, (B) male individuals carrying *SMARCA1*variants (SMARCA1_M), and (C) female individuals with *SMARCA1* variants (SMARCA1_F). MDS plots (center) and heatmaps (right) with Euclidean clustering show clustering of cases (red for males, orange for females) and unaffected controls (blue).Columns of the heatmap correspond to the samples and rows correspond to the selected probes.

**Figure 4:**
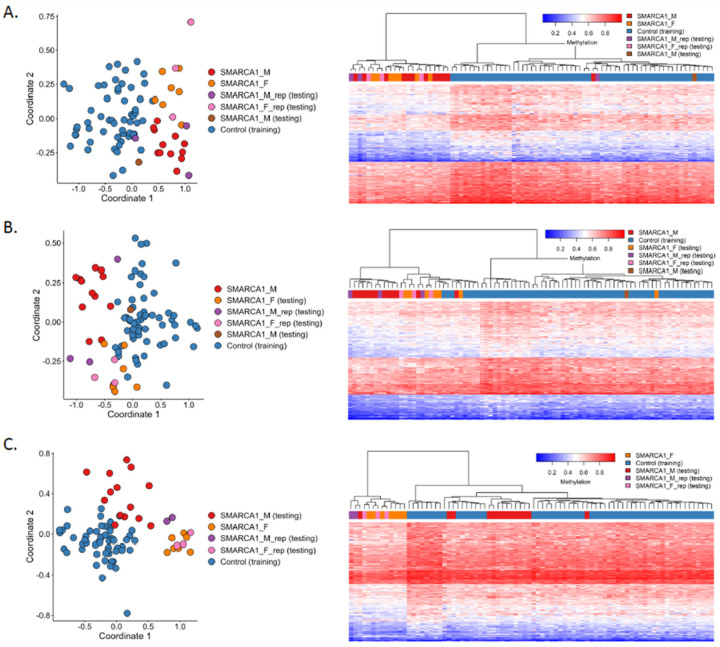
Unsupervised clustering for non-discovery samples using methylation probes for *SMARCA1* Cohort. Other *SMARCA1* samples not used in *SMARCA1* profile discovery were investigated using unsupervised analysis and examined using the distinct profiles for (A) SMARCA1 cohort, (B) SMARCA1_M cohort, and (C) SMARCA1_F cohort. As expected, replicates cluster with respective similar cases for each profile.

Initial analysis of all 19 individuals compared to 57 controls matched by age, sex and array type revealed a predominantly hypomethylated profile defined by 232 probes differentiating cases from controls (Figure 3A). LB replicates also exhibited the same methylation pattern, while the additional test SMARCA1_M sample exhibited a pattern more similar to controls (Figure 4A). Gender-specific methylation profiles were also observed. SMARCA1_M samples matched to 60 unaffected controls showed a generally hypomethylated pattern for 212 probes, and the SMARCA1_F samples matched to 56 unaffected controls also displayed lower methylation levels for 221 distinct probes. The two probe sets have absolute methylation difference ranges 5–13% and 5–16%, respectively, and distinguish the SMARCA1 cases from the matched controls (Figure 3B–C). While the initial global methylation profile does not separate the individual gender sub-cohorts, the profiles for each gender were able to distinguish between them up to a certain degree. The SMARCA1_F samples and replicates mostly cluster with SMARCA1_M cases but in a separate sub-cluster with a subset of the controls in the heatmap, and majority of the SMARCA1_M samples and replicates also matched the methylation pattern of the controls evident in the heatmaps Figure 4B and Figure 4C, respectively. However, MDS plots reveal some level of separation between the male samples, female samples, and unaffected samples.

### Characterization of Smarca1 conditional knockout mice

Similar to a subset of the *SMARCA1* cohort, we previously reported that an in frame-deletion of exon 6 in the murine *Smarca1* gene (Ex6DEL mice) resulted in animals with an enlarged brain phenotype (38). The Ex6DEL mice generate an internally truncated Smarca1 protein that was unable to hydrolyze ATP but retained the ability to bind its interaction partners thereby suggesting it functions as a dominant negative protein (49). Since most individuals with macrocephaly in the *SMARCA1* cohort contained LOF variants, it raised more general questions about the pathogenic nature of different *SMARCA1* variants and whether *SMARCA5* inclusion within the NURF complex also mitigated pathogenicity in some individuals.

**Figure 5:**
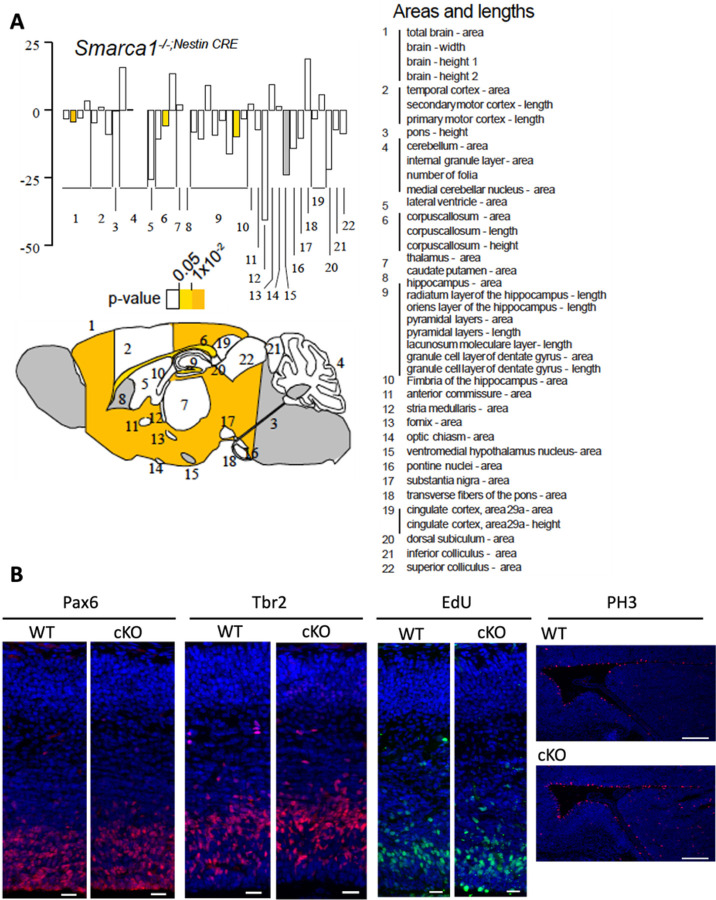
Smarca1 cKO mice have minor changes in brain size. A) Schematic illustration of a mouse saggital brain section (bottom). Numbers identify the different brain regions analyzed (right) for area and length differences between *Smarca1* cKO and control littermates. Graph (top) depicts the size changes observed. The light and dark yellow shading highlights the statistically significant differences (p<0.05 and p<0.01, respectively) while the grey shading denotes regions not analyzed for this study. B) Control (WT) and *Smarca1* cKO (cKO) coronal brain sections isolated at E14.5 were IF-stained to detect radial glia progenitors (Pax6), intermediate progenitors (Tbr2), or mitotic progenitor cells (PH3). An EdU-click chemistry reaction was used to identify progenitor cells in S-phase. Cell nuclei were counter-stained with Hoechst dye (blue). Images were taken at the dorsomedial region of the developing cortex. Marker-positive cells were quantified as described in the text and shown in Supplemental Figure 4. Scale bars = 100 mm for PH3 and 20 mm for all other panels.

**Figure 6:**
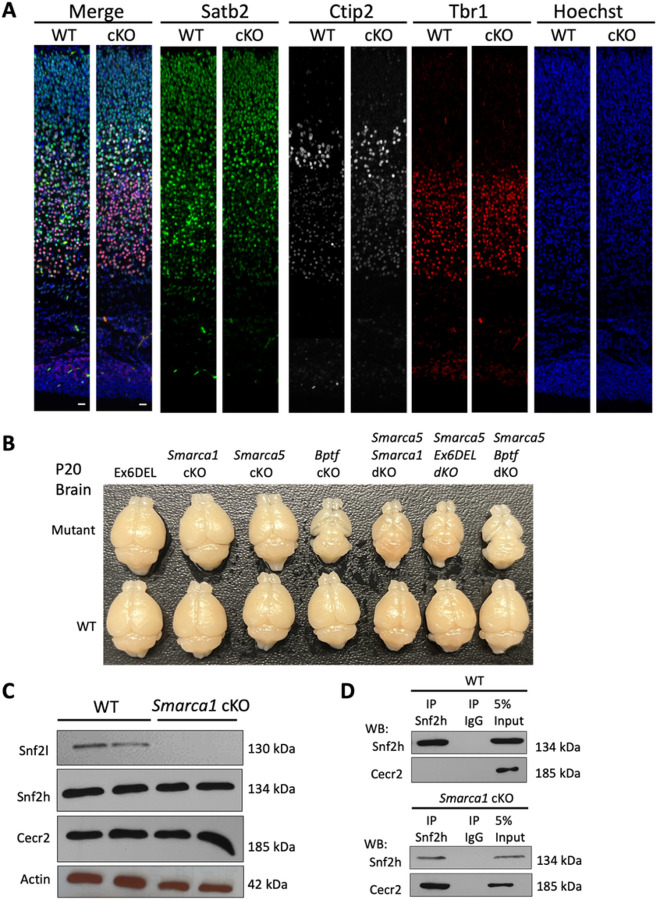
Variations in cortical growth caused by ablation of different NURF components. A) Control (WT) and *Smarca1* cKO (cKO) coronal brain sections isolated at P0 and imaged at the dorsomedial region of the cerebral cortex. Sections were IF-stained for the deep layer neuronal markers Tbr1 (red; layer VI) and Ctip2 (white; layer V), and the upper layer neuronal marker Satb2 (green, layers II-V). The merged images are shown in the left panels. Cell nuclei were counter-stained with Hoechst dye (blue; right panels). Scale bars = 20 mm. B) Photographs of P20 brains dissected from transgenic mice corresponding to the genetic ablation of different genes that comprise the NURF complex (mutant) or from their control littermates (WT). Ex6DEL refers to mice with a deletion of exon 6 of the *Smarca1* gene that generates an internally truncated Snf2l protein lacking part of the ATPase/helicase domain. cKO, conditional knockout; dKO, double conditional knockout. C) The CERF complex, comprising Snf2l and Cecr2, is abundantly expressed in the cerebellum. P10 cerebellar extracts were isolated from control (WT) or *Smarca1* cKO animals and used for immunoblots to examine CERF protein levels and Snf2h abundance. D) Co-immunoprecipitation assays were performed from WT (upper panels) or *Smarca1*cKO (lower panels) cerebellar extracts with antibodies to Snf2h or rabbit IgG. Snf2h antibodies immunoprecipitated Cecr2 only when Snf2l protein was absent (*Smarca1*cKO). 5% of the total IP lysate was loaded as input.

To model the LOF variants, we obtained *Smarca1* targeted ES cells from the UC Davis Knockout Mouse Project (KOMP) repository (MMRRC:062622-UCD; *Smarca1*^tm1a(KOMP)Wtsi^) in which exon 12 was flanked with loxP sites. Introduction of Cre recombinase results in deletion of exon 12 and generates an out-of-frame transcript and a LOF variant. Conditional hemizygous knockout male animals (referred herein as *Smarca1* cKO) were generated by crossing the *Smarca1*^tm1a(KOMP)Wtsi^ mice with a nestin-Cre driver line for selective deletion in neuronal cells (33). *Smarca1* cKO mice were viable and born in normal Mendelian ratios with no obvious gross motor, behavioral, or morphological deficits. Brains dissected from wild type (WT) and *Smarca1* cKO adult mice were collected and examined for neuroanatomical differences in 22 distinct brain regions, as described previously (50). The *Smarca1* cKO brains showed little difference from WT littermates although slight size reductions were observed in total brain width, corpus callosum length, and the area of the granule cell layer of the dentate gyrus (Figure 5A). Within the E15.5 developing neocortex we observed no differences in the proportion of radial glial (Pax6+) or intermediate (Tbr2+) neural progenitor cells (Figure 5B; Suppl. Figure 2). Embryonic brains were pulsed for one hour with EdU to label progenitor cells in S-phase, which also showed no deficit in the proliferating proportion of the two progenitor populations. Staining with phospho-histone H3 (PH3), a marker of mitotic cells revealed an increase in the total number of PH3+ cells along the length of the lateral ventricle in the *Smarca1* cKO animals, yet this had no impact on overall cell number in the developing cortex (Figure 5B; Suppl. Figure 2). Cortical lamination was examined by immunostaining postnatal day 0 (P0) brain sections for deep layer (Tbr1, Ctip2) and upper layer (Satb2) neuronal markers. *Smarca1* cKO and WT brain sections at P0 showed no deficits in cortical lamination (Figure 6A; Suppl. Figure 2). Taken together, we conclude that the *Smarca1* cKO mice do not present with a brain overgrowth phenotype or with major deficits in forebrain development, which is in stark contrast to the phenotype of the Ex6DEL mice and many individuals of the *SMARCA1* cohort with LOF variants.

Since the ISWI subunits are interchangeable within the NURF complex (12), the severe microcephaly observed in *Smarca5* and *Bptf* conditional knockouts also suggest that *Smarca1* and *Smarca5* may regulate brain growth via different mechanisms. To determine how specific NURF complexes relate to cortical growth we examined brain size from age-matched mice containing single, or double gene knockouts of the *Smarca1, Smarca5*, or *Bptf* genes that comprise the NURF complex. The comparison showed that for the single gene knockouts, the *Bptf* knockout animals (*Bptf* cKO) had the most severe cortical phenotype (Figure 6B), followed by the Smarca5 mutants which matched previous reports (36, 51). Moreover, all combinations of double mutants (*Smarca1/Smarca5* dKO, *Smarca5*/Ex6DEL, and *Smarca5/Bptf* dKO) resulted in a phenotype similar to the *Bptf* mutants alone (Figure 6B). Surprisingly, the enlarged brain of the Ex6DEL mice was reduced to the same extent as the other NURF mutants when combined with *Smarca5* ablation. Taken together, these data reveal that proper cortical development requires NURF, and that *Smarca5* compensates considerably for *Smarca1* mutations.

The less severe hypoplastic neocortex of the *Smarca5* cKO mice compared to the *Bptf* cKO animals likely results from the reported up-regulation of *Smarca1* (33, 36). To determine if *Smarca5* upregulation and a corresponding Snf2h protein increase may similarly compensate for *Smarca1* gene ablation, we examined the formation and composition of the CERF complex (52) within the cerebellum of WT and *Smarca1* cKO mice. The switch to the cerebellum and analysis of the CERF complex was chosen for three reasons: 1) the lack of commercially available antibodies specific to Bptf for analysis of the NURF complex; 2) the *Smarca5* cKO and Ex6DEL mutant animals show similar differential effects on cerebellar size to those observed in the neocortex; and 3) the CERF complex is enriched in the cerebellum (33, 49). We observed that Snf2h and Cecr2 proteins were not upregulated but expressed at comparable levels in the WT and *Smarca1* cKO cerebellar extracts isolated from P21 mice (Figure 6C). Immunoblots also confirmed that Snf2l protein was absent in the *Smarca1* cKO samples (Figure 6C). Using Snf2h antibodies for immunoprecipitation (IP) assays, Cecr2 was not co-purified in the WT samples (Figure 6D, top panel) suggesting that the CERF complex comprised only Snf2l at P21, consistent with our previous findings (49). However, in *Smarca1* cKO lysates the Snf2h antibody was able to co-IP the Cecr2 protein suggesting that it functionally compensates for the loss of Snf2l protein (Figure 6D, lower panel).

## Discussion

We describe 41 individuals with a novel and variable neurodevelopmental syndrome caused by both LOF and missense variants in the X-linked *SMARCA1* gene. Including the index family, a total of 10 different nonsense or frameshifting variants were identified that provide strong evidence of disease causation for the *SMARCA1* gene. Three quarters of the affected individuals described were hemizygous males, most with maternally inherited variants. Hemizygous females comprised the remaining 25% of affected cases and these individuals often showed a milder phenotype. The most prevalent clinical findings were ID/DD (80%) and speech delay or speech regression (85%) while other phenotypic features were more variable. Affected individuals were stratified into three groups based on mutation type: males with LOF mutations, males with missense mutations, and females with phenotypic features.

The index family carried a nonsense variant (p.Arg91*) that resulted in a brain overgrowth feature, the penetrance of which was nearly complete for all 10 males with LOF variants. Three exceptions were observed, namely p.Leu357*, p.Arg1035Glnfs*13, and the previously reported p.Gln3*. Of the exceptions, it is possible that the p.Gln3* and the p.Arg1035Glnfs*13 variants generate stable truncated proteins resulting from the use of an alternative Met start codon (p.Gln*), or from a transcript that escapes nonsense mediated decay (p.Arg1035Glnfs*13), respectively. Nonetheless, the p.Leu357* variant remains a phenotypic outlier for brain overgrowth. Moreover, the brain overgrowth genotype/phenotype correlation was not restricted to truncating variants as five males with missense variants (p.Thr118Ile, p.Gly771Lys, p.Asn778Asp, p.Pro823Leu, and p.Phe1003Leu) also presented with enlarged brain size. To further stratify the phenotype, we generated genome-wide methylation profiles which have been described in a growing number of genetic conditions including in the SWI/SNF remodeling complex disorders and can be used to develop sensitive and specific molecular biomarkers referred to as epi-signatures (53, 54). While we demonstrated evidence of a mild global DNA methylation profile shared by patients with truncating variants and overgrowth, these changes are not sufficiently robust to be considered an epi-signature at this time. Our ongoing work in expanding the DNA methylation data set may enable development of a sensitive biomarker in the future.

The second cohort of affected individuals comprised 19 males with *SMARCA1* missense variants with variable neurodevelopmental features and aside from the 5 individuals described above, they presented with a normal sized brain. These variants were located throughout the gene and for the most part no specific genotype/phenotype correlations were delineated. Affected individuals from three families and one previously reported family (39) were found to carry the recurrent p.Val384Met variant. While the pathogenicity of the p.Val384Met variant remains questionable, an affected male from family 16 suffered from bipolar disorder, psychosis, ADHD, and suicidal ideations. His mother, who was heterozygous for the variant also had a diagnosis of psychosis and bipolar disorder. These phenotypic features were similar to the previously published family with schizophrenia in which four affected siblings and their unaffected mother also had the p.Val384Met variant (39). One other individual carrying the p.Ile647Thr variant also developed psychotic symptoms suggesting that *SMARCA1* is involved in the biological pathways implicated in schizophrenia.

The third cohort comprised 9 affected females of which 7 contained LOF alleles. Within the index family, individuals V2 (44 yrs) and V10 (48 yrs) had no reported learning disability and, although head circumference was mildly increased (+1.0 and +1.5 SD, respectively), macrocephaly and overgrowth were not significant. While this suggests that milder features of the disease may be present in females, there was sparse clinical data available from mothers of affected individuals. In addition, X-inactivation studies were available from three females only and they were discordant with phenotype. As such, further investigation is required to fully assess whether X-inactivation skewing may modulate the phenotype in affected females.

*SMARCA1* joins the *BPTF* and *SMARCA5* genes as members of the ISWI family of remodeling complexes that are causative of NDDs (37, 46). The latter two syndromes show considerable phenotypic overlap with microcephaly, a similar facial gestalt, and sandal gap toes common to each syndrome. Like the human *BPTF* and *SMARCA5* NDDs, microcephaly is a common feature of the forebrain-specific conditional knockout mice for the *Smarca5* and *Bptf* genes (36, 51). Both *Smarca5* and *Bptf* mouse mutants show a reduction in intermediate progenitors, suggesting that the NURF complex has a critical role in amplifying this progenitor pool. Indeed, *Bptf* seems to be the critical determinant since none of the double knockouts (*Smarca1/Smarca5*, *Smarca5*/Ex6DEL, or *Smarca5/Bptf*) were any more severe than *Bptf* cKO alone (Figure 6b).

Individuals with pathogenic variants in *SMARCA1* are phenotypically distinct in comparison to *BPTF* and *SMARCA5* NDDs, as suggested primarily by the high penetrance of macrocephaly. The opposite effect of pathogenic *SMARCA1* versus *SMARCA5* variants on cortical growth might be explained by the fact that SNF2H functions in proliferating progenitors while SNF2L drives differentiation. Indeed, data from our genetic epistasis experiments provide *in vivo* evidence for the phenotypic differences observed in individuals with pathogenic variants in these closely related genes. First, in the absence of *Smarca5*, we observed an upregulation of *Smarca1* that presumably provides some compensation during progenitor proliferation as the cortical dysplasia in these animals is not as severe as that observed in the *Bptf* mutant mice (33, 36). Second, when *Smarca1* is ablated we observed only minor differences in brain size and no significant changes in cortical development (Figures 5A, B, and 6A, B) suggesting that in mice *Smarca5* can adequately compensate for *Smarca1* loss during corticogenesis. This finding was further supported by our demonstration that Snf2h protein can complex with Cecr2 in the absence of Snf2l protein but not in control samples where Snf2l is abundant (Figure 6D). Moreover, this data is the first *in vivo* demonstration that the ISWI subunits are interchangeable within a complex.

The internally truncated Snf2l protein in the Ex6DEL mice is unable to bind or hydrolyze ATP, and despite associating with partner proteins the neural progenitors showed prolonged chromatin accessibility at a subclass of promoters that delayed differentiation and enhanced proliferation (38, 49). These dominant negative effects resulted in the enlarged brain size of the Ex6DEL mice which was also observed in most LOF human *SMARCA1* variants. In this scenario, as Snf2h protein levels decrease, the NURF complex switches Snf2l to promote neuronal differentiation. However, the retention of a catalytically inactive Snf2l protein impairs proper differentiation and effectively blocks any compensatory effects by residual Snf2h-NURF complexes. In this regard, individuals with macrocephaly that carry missense variants would be predicted to encode proteins with dominant negative effects - incorporating into complexes but lacking chromatin remodeling activity. Four of the five missense variants associated with brain overgrowth lie within the HSS domain. The HSS domain interacts with the linker DNA located between nucleosomes and is mechanistically critical to the DNA translocation around the nucleosome (55). Nonetheless, the macrocephaly observed in individuals with LOF variants in *SMARCA1* remain challenging to explain with this argument, unless some of these LOF variants escape NMD and behave in a dominant negative manner. Otherwise, if the LOF variants undergo NMD we would predict that SNF2H would be retained within NURF resulting in normal brain growth – as per the observations in the *Smarca1* cKO mice. An alternate explanation is that, in humans, SNF2H cannot compensate for the loss of SNF2L protein in the intermediate progenitor cells during forebrain development leading to a distinct phenotype.

**Figure 7:**
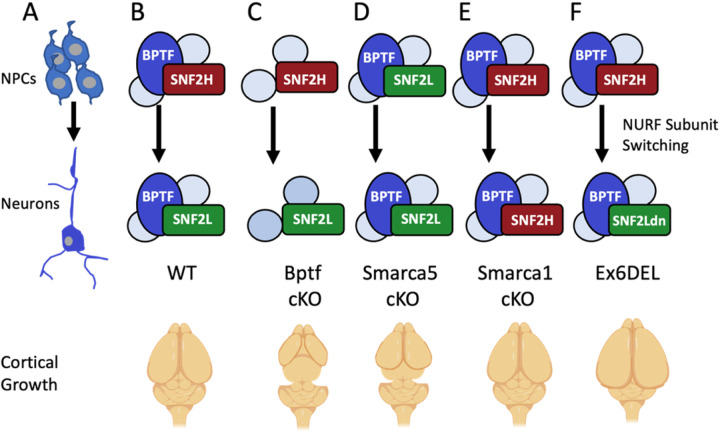
Normal and aberrant NURF subunit switching during mouse cortical development. A) The NURF complex is expressed in proliferating neuroprogenitors (NPCs) and in differentiated neurons during murine corticogenesis. B) During normal cortical development (WT) the catalytic subunit within NURF during growth is SNF2H (encoded by SMARCA5). During differentiation, the catalytic subunit is switched to SNF2L (encoded by *SMARCA1*). C) Ablation of the *Bptf* gene (*Bptf* cKO) prevents formation of the NURF complex and results in minimal growth and a severely hypoplastic cortex. Individuals with BPTF pathogenic variants also present with microcephaly. D) When *Smarca5* is inactivated (Smarca5 cKO), there is some compensation by SNF2L during growth that results in a smaller cortex but one that is slightly larger than the *Bptf* cKO mice. Individuals with *SMARCA5* variants also present with microcephaly. E) *Smarca1* knockout mice (*Smarca1*cKO) have a normal sized cortex which results from near complete compensation by the SNF2H protein. Individuals with *SMARCA1* nonsense LOF variants have a high penetrance of macrocephaly, which is discordant from the mouse phenotype. F) In the Ex6DEL mice subunit switching occurs normally but the mutant protein lacks remodeling activity creating a dominant negative effect that delays differentiation and results in a larger brain size. Individuals with *SMARCA1* variants and macrocephaly are predicted to lack remodeling activity and function in a dominant negative fashion while other missense variants that don’t abolish remodeling activity are not macrocephalic.

Finally, individuals with missense variants in the ATPase or HSS domains that do not present with macrocephaly are likely functional hypomorphs that retain some chromatin binding and remodeling activity. Certainly, additional work is necessary to define the specific effects of different variants on SNF2L function during neurogenesis. Furthermore, an examination of SNF2H and/or BPTF protein levels in individuals with *SMARCA1* pathogenic variants may also prove interesting in defining how they modulate phenotypic variability. As summarized in Figure 7 for mice, the interplay between SNF2H, SNF2L, and BPTF protein levels, their temporal and spatial association within the NURF complex, and how that dictates its genomic targets are critical factors for defining brain size and, more importantly, disease severity. Given that a clinical continuum is observed in Coffin-Siris and Nicolaides-Baraitser syndromes depending on which BAF encoding gene is mutated (3, 53), it is not unusual to consider that *SMARCA1, SMARCA5*, and *BPTF* NDDs may also align along a clinical spectrum.

## Materials and Methods

### Cohort ascertainment

Pathogenic variants were identified in *SMARCA1* using a combinationof trio-based exome sequencing (ES; 23 families), short read whole genome sequencing (WGS; 3 families) in both clinical diagnostic and research settings. Families were identified across several institutions with data shared via nodes of the MatchMaker Exchange (MME) network, including MyGene2, Gene-Matcher, PhenomeCentral, and by querying investigators with large cohorts of patients with intellectual disability, macrocephaly, overgrowth and/or other neurodevelopmental features (47). All available clinical data from affected individuals were reviewed by the investigators. The study was approved by Seattle Children’s Hospital Institutional Review Board (IRB# 13921). All participants or their legal guardians signed an informed consent for publication of clinical and genotype data according to the Declaration of Helsinki or were de-identified and included in the study under an IRB approved ‘waiver of consent’. Additional permission for publication of photographs were obtained by from participants or their legal guardians using standard forms at each local site by the responsible referring physicians.

### Methylation data analysis

The *SMARCA1* cohort for methylation analysis consists of 19 individuals from 9 families: 7 females and 12 males for whom samples were available. Among the cohort, four individuals are unaffected female carriers of *SMARCA1* variants, 13 carry truncating variants and 6 have missense variants (See Table 1). DNA samples derived from peripheral blood were collected and anonymized. Replicate samples were also obtained from 6 individuals, 5 using LB-line and 1 using peripheral blood and were only used for investigating unsupervised clustering using the identified probes for the methylation profiles.

The DNA obtained from the samples collected were subjected to bisulfite conversion using Illumina Infinium MethylationEPIC BeadChip kit. The manufacturer’s protocols were followed and quality control of the generated methylation data was done using the minfi R package (56). Standard data preprocessing for Illumina arrays were implemented, including background correction and normalization. Density plots of beta values were checked, as well as recorded and predicted gender and age differences. Analysis was done on the pre-processed methylation data as described in previous research (57–59). Initial filtering of probes includes removing those with failed detected p-values, are located in the X or Y chromosome, cross-reactive, or known to target CpG sites overlapping SNPs. This resulted to 772557 probes before the main analysis.

For differential methylation analysis, matched unaffected controls were first selected from the London Health Sciences Centre EpiSign Knowledge Database (EKD) (57–59). Batches in the EKD known to cause batch effects and samples with more than 5% failed probes were excluded. Selection was done by matching age, sex and array type of the cases using the R MatchIt package (60) with case-control ratios ranging from 1:3 to 1:8, depending on the number of cases in the analysis. Batch effects and outliers in the original cohort as well as the case-control training data used for discovery of differential profiles were inspected using principal component analysis (PCA).

Differential analysis of methylation signals between cases and matched controls was implemented for defining methylation profiles. A linear regression model was fitted using the limma R package (61), with the methylation M values as predictors and case/control labels as response. Covariates were also included using the estimated blood cell proportions of the samples. Using empirical Bayes method, moderated t-statistics and corresponding p-values were computed for each probe, and p-values were adjusted using the Benjamini-Hochberg method to control for false discovery rates. Methylation differences were also estimated using beta values. Different parameter combinations involving the ranks by p-value and variable importance using receiver operating characteristic (ROC) curve analysis, as well as correlation, were considered for probes with minimum methylation difference of 5% between cases and controls. The separation of the two groups was examined using hierarchical clustering and multidimensional scaling (MDS). A metric was also formulated to assess quality of clustering by considering the minimum distance between the two groups and their respective standard deviations. The final parameter values and the selected differentially methylated probes defining the methylation profile were determined using the best clustering score.

### Mouse generation and animal husbandry

The study was approved by the University of Ottawa’s Animal Care ethics committee (Protocol # OHRI-3762 and OHRI-3773) and meets the standards set by the Canadian Council on Animal Care and by the Animal Care and Veterinary Services (ACVS) facility of the University of Ottawa. Animals were housed in a facility under SPF (specific pathogen-free) conditions on a 12/12 light:dark cycle with water and food ad *libitum*.All experiments were performed according to the guidelines set by the University of Ottawa’s Animal Care ethics committee, maintaining the standards set by the Canadian Council on Animal Care in the Animal Care and Veterinary Services (ACVS) facility of the University of Ottawa. The Ex6DEL, *Smarca5* cKO, *Bptf* cKO, and Emx1-Cre mouse lines and genotyping protocols have been described previously (33, 36, 38, 51, 62). *Smarca1* engineered ESCs obtained from the KOMP repository (MMRRC:062622-UCD; Smarca1^tm1a(KOMP)Wtsi^) were passaged for 7 days then harvested and provided to the University of Ottawa Transgenic Mouse Core Facility for blastocyst injections using a previously described protocol (63). Six chimeric mice were identified, four showed germline transmission when bred to C57BL/6 female mice and these were bred to homozygosity to generate the *Smarca1*^f/f^ line. To obtain *Smarca1* cKO mice, *Smarca1*^f/f^ females were bred to the Nestin-Cre line described previously (64). Double knockout mice were generated by interbreeding f/f lines prior to breeding to the Nestin-Cre or Emx1-Cre driver line.

Genotyping of the Smarca1 cKO mice was performed under the following PCR conditions: a denaturing cycle at 94°C for 2 min, 35 PCR cycles (94°C for 20 sec, 63°C for 25 sec, 72°C for 25 sec) and a final cycle at 72°C for 1 min with the following primer sequences: *CSD-loxF* (5’-GAGATGGCGCAACGCAATTAATG -3’) and CSD-Smarca1-R (5’-AAGAACACACTGGGTGCTAGGTAGG-3’) to amplify the Snf2l flox allele (350 bp product); and CSD-Smarca1 (5’-CCCCTCAGAGGACAGTTATGCTAGG-3’) and CSD-Smarca1-ttR (5’-GCAGACATCATGAATCTTGCAGGC -3’) to amplify the Snf2l WT allele (602 bp product).

### Tissue collection, EdU injection, and neuroanatomical analysis

To obtain embryonic brains, time-mated mice were established and checked daily for a vaginal plug, which was considered embryonic day 0.5 (E0.5) of the gestation period. At E15.5, embryos were quickly dissected, heads cut off and fixed in 4% paraformaldehyde (PFA) at 4° C overnight. For EdU pulse labeling, timed-mated pregnant females were injected intraperitoneally with 100 mg/g body weight of 5-Ethynyl-20-deoxyuridine (EdU; Sigma-Aldrich, Oakville, ON, Canada) 60 min before sacrifice and tissue collection. The following day, heads were placed in a 30% sucrose solution and once submerged they were transferred to a 1:1 solution of 30% sucrose solution and OCT (Tissue-Tek, Sakura Americas, Torrance, CA, United States) then snap-frozen on liquid nitrogen and stored at −80°C until sectioned. Brains analyzed at postnatal day 0.5 (P0.5) were prepared in the same manner as embryonic brains. P20 mice were transcardially perfused with saline followed by 4% paraformaldehyde (PFA) in 0.1 M PBS, prior to dissecting the brains. Brains were incubated in 4% PFA at 4° C overnight and the remainder of the protocol was identical to the embryonic brain protocol described above. The protocol used for 2D-neuroanatomical measurements of *Smarca1* cKO brains has been described in detail (50).

### Immunofluorescence

Frozen murine brains embedded in OCT were sectioned (12 μm) using a Leica CM1850 cryostat and then washed in 1xPBST (0.1M Phosphate-Buffered Saline, 0.1% Triton X-100) before use. Where antigen retrieval was required, slides were incubated in 10 mM Sodium citrate buffer (pH 6.0) that was heated to 80° C for 30 min. For EdU immunodetection, sections were first washed with 1X PBS prior to incubation with an EdU staining solution (100 mM Tris-HCL pH 7.2, 2 mM CuSO4, 10 mM fluorescent azide, 50 mM ascorbic acid) for 1 hour at room temperature. Slides were then washed and incubated with Hoechst dye before imaging as described below.

Sections were blocked (10% horse serum/PBST) for 30 min at room temperature. All slides were then incubated overnight at 4° C in primary antibody solution (0.04% Triton X-100, 3 mg/mL bovine serum albumin in 1X PBS) with the following primary antibodies: rabbit anti-Pax6 (1:500; Cedarlane, PRB-278P-100); rabbit anti-Tbr2 (1:200; Abcam, ab23345); rabbit anti-Tbr1 (1:100; Abcam, ab31940); rat anti-Ctip2 (1:300; Sigma, 06–570); mouse anti-Satb2 (1:300; Abcam, ab51502); and rabbit anti-phospho-Histone H3 (1:500, Abcam, ab12345). Slides were then washed in 1xPBS and incubated for 1 hour at room temperature with a 1:500 dilution of the appropriate secondary antibody. Secondary antibodies included anti-rabbit-647 Alex Fluor (Invitrogen, a31573), anti-mouse-488 Alex Fluor (Invitrogen, a21202), anti-rat-555 (Invitrogen, a48263), or anti-rabbit-555 (Invitrogen, a31572). Sections were then washed several times with 1xPBS, incubated with Hoechst 33342 dye (1 mg/ml; 20-[4-ethoxyphenyl]-5-[4-methyl-1-piperazinyl]-2,50-bi-1H-benzimidazole trihydrochloride trihydrate; Thermo FisherScientific, #H3570) for 15 min at room temperature. Finally, slides were mounted on coverslips with DAKO fluorescent mounting medium (Agilent Technologies, #S3023) and imaged with a Zeiss Axiovert Observer Z1 epifluorescent/light microscope with an AxioCam cooled color camera (Zeiss). Images were exported to Adobe Photoshop CS5 (Adobe Systems, Inc.) for further processing, cell counting, and figure preparation.

Cell counts were performed from the coronal cortical images acquired, using a representative region demarcated with a 200 mm wide column that comprised the full height of the cortex. Within each column, the mean cell number (any marker positive cell) and total cell count (Hoechst+ nuclei) was quantified from a minimum of three sections from at least three biological replicates. A student t-test was used for statistical comparison between control and *Smarca1* cKO samples. Resulting bar graph plots denote mean values +/− SEM. An asterisk was used to designate statistical significance (p < 0.05).

### Protein isolation, immunoblots and co-immunoprecipitation

Brain cortices or cerebella were quickly dissected from individual animals and snap frozen in liquid nitrogen. Tissues were manually sheared then incubated at 4° C, with rocking, in ice-cold lysis buffer (20 mM Tris-HCl pH 8, 137 mM NaCl, 1% NP-40, 2 mM EDTA) supplemented with Halt protease inhibitor cocktail (Thermo Fisher Scientific, 78425) for 10 min. Lysed samples were pre-cleared by centrifugation (12,000 x g for 15 min) and proteins quantified using the Bio-Rad Protein Assay Dye Reagent Concentrate (Bio-Rad Laboratories). Protein samples were resolved on Bis-Tris 4–12% gradient gels (NuPage, Invitrogen, USA) by electrophoresis (90–150 V) using the Bio-Rad Mini-PROTEAN Tetra Cell and then blotted onto PVDF membranes (Immobilon-P; Millipore) by wet transfer at 0.35 A for 90 mins using the Bio-Rad Mini Trans-Blot cell. Membranes were blocked (45 min, room temperature) with 5% skim milk in TBST (Tris-buffered saline containing 0.05% Triton X-100), and incubated (4°C, overnight) with the following antibodies: : rabbit anti-SNF2H (1:500; Abcam ab72499); rabbit anti-SNF2L (1:500; Abcam ab37003); rabbit anti-CECR2 (1:500; gift from Dr. Heather McDermid, Univ. Alberta); mouse anti-b-actin (1:3,000; Sigma, A1978). After washing (5x 5 min TBST) the membranes were incubated for 1 hour at room temperature with HRP-conjugated goat anti-rabbit (1:5000, Jackson Immunoresearch, 111–035-003) IgG (H+L) secondary antibodies. Membranes were washed 5 x 5 min in TBST after antibody incubations, and signals were detected using the Pierce Supersignal West Fempto (Pierce, Cat # 34095) chemiluminescence substrate.

For co-immunoprecipitation, each reaction consisted of 500 mg of protein lysate, with 1 mg of Snf2h antibody (Abcam, ab72499) or rabbit IgG for negative controls. The final volume was adjusted to 500 ml with protease inhibitor (1/1000) and lysis buffer. Reactions were incubated at 4 °C overnight and then 30 ml of Protein A/G magnetic beads (Bioclone Inc, cat # MA102) were added and the incubation continued for another hour. Beads were washed 5 times (at 4 °C in 5 min intervals) in PBS containing 0.3% triton-X before eluting in 0.1M glycine (pH 2.5) at room temperature for 10 min, with occasional agitation. Elution step was performed 4 times and volumes pooled prior to immunoblotting (described above).

## Supplementary Material

Supplement 1
