# Supplementary material for: Pathogenic variants in SMARCA1 cause an X-linked neurodevelopmental disorder modulated by NURF complex composition
Source: Res Sq. 2023 Sep 29:rs.3.rs-3317938. Preprint. [Version 1] doi: 10.21203/rs.3.rs-3317938/v1 (PMC10571636; doi:10.21203/rs.3.rs-3317938/v1)
Supplement: Supplement 1 [file NIHPPrs3317938v1-supplement-1.pdf]

Supplementary Files

This is a list of supplementary files associated with this preprint. Click to download.

- MirzaaetalSupplInfo.pdf
